# Design and Usability Testing of a Novel Internet-Delivered Cognitive Behavioral Therapy (iCBT) Software Platform for Children with Anxiety

**DOI:** 10.3390/children12111535

**Published:** 2025-11-13

**Authors:** Maria Carmela Pera, Caterina Poli, Martina Gnazzo, Valentina Baldini, Laura Delsante, Marco Pacchioni, Mirko Orsini, Beatrice Rita Campana, Francesca Diodati, Matteo Puntoni, Giuseppe Maglietta, Caterina Caminiti, Susanna Esposito

**Affiliations:** 1 Pediatric Clinic, Department of Medicine and Surgery, University of Parma, 43126 Parma, Italy; mariacarmela.pera@unipr.it (M.C.P.); dottoressapolic@gmail.com (C.P.); beatricerita.campana@unipr.it (B.R.C.); 2 Department of Biomedical, Metabolic and Neural Sciences, University of Modena and Reggio Emilia, 41125 Modena, Italy; martinagnazzo8@gmail.com (M.G.); valeria.baldini@unimore.it (V.B.); 3 Department of Biomedical and Neuromotor Sciences, University of Bologna, 40127 Bologna, Italy; 4 DataRiver Srl, 41122 Modena, Italy; laura.delsante@datariver.it (L.D.); marco.pacchioni@datariver.it (M.P.); mirko.orsini@datariver.it (M.O.); 5 Clinical and Epidemiological Research Unit, University-Hospital of Parma, 43126 Parma, Italy; fdiodati@ao.pr.it (F.D.); mpuntoni@ao.pr.it (M.P.); gmaglietta@ao.pr.it (G.M.); ccaminiti@ao.pr.it (C.C.)

**Keywords:** internet-based cognitive behavioral therapy, anxiety, children, digital therapeutics, mHealth, gamification

## Abstract

*Background:* Anxiety disorders are common in childhood, yet access to cognitive behavioral therapy (CBT) is often limited. Internet-delivered CBT (iCBT) can help overcome these barriers, but evidence in younger children remains scarce. This pilot study describes the development and preliminary evaluation of an Italian iCBT platform for children with mild to moderate anxiety. *Methods:* Five children aged 8–12 years and their caregivers were recruited through pediatricians. Eligibility was assessed using the MASC-2 and a psychiatrist interview. Each child completed a supervised session with the WebApp, which delivers CBT modules combining psychoeducation, cognitive restructuring, relaxation, and gamified activities. Usability was evaluated using the ita-MAUQ, observation, and interviews. *Results:* All participants completed the session without dropouts. Mean ita-MAUQ scores were consistently above the midpoint, with the highest ratings for interface design and satisfaction. Children appreciated the interactive, game-like features, while caregivers valued the clarity and practicality of content. Qualitative feedback indicated good comprehensibility and engagement, with suggestions for improving navigation flow and language adaptation. No adverse events occurred. *Conclusions:* This pilot study supports the feasibility, safety, and acceptability of the new iCBT platform and provides essential insights for its refinement and future large-scale clinical trials.

## 1. Introduction

Anxiety disorders are among the most prevalent psychiatric conditions in children and adolescents, affecting approximately 6–7% of the global population [1,2,3]. While anxiety and fear are universal developmental experiences that typically resolve with time, in some children, these responses become disproportionate to the stimulus and inconsistent with developmental stage, resulting in an anxiety disorder [4].

Early-onset anxiety is linked to numerous adverse outcomes, including reduced academic performance, difficulties in peer relationships, and an increased risk of persistent mental health disorders in adulthood [5,6]. The impact extends beyond the child, affecting family functioning and caregiver well-being. Active involvement of parents is therefore essential, as it fosters awareness, discourages protective behaviors such as avoidance, and promotes the reinforcement of adaptive coping strategies [7].

Although evidence-based treatments such as cognitive behavioral therapy (CBT) are available, the majority of affected children do not receive appropriate care [8,9]. CBT has consistently demonstrated efficacy across pediatric and adult anxiety disorders [10,11], and early intervention is particularly valuable given the heightened neuroplasticity of the developing brain, which enhances long-term therapeutic outcomes [7].

Significant barriers, including limited availability of trained clinicians, long waitlists, geographic constraints, and stigma, contribute to the treatment gap—especially in underserved settings [12]. Digital therapeutics (DTx), defined as software-based interventions designed to prevent, manage, or treat medical conditions, represent a promising avenue to improve access. Unlike general wellness applications, DTx are clinically validated, regulated, and often delivered under professional supervision, aligning with international standards for medical devices [13,14,15]. Among these, internet-delivered CBT (iCBT) has emerged as a scalable modality that preserves the key therapeutic components of traditional CBT—psychoeducation, cognitive restructuring, exposure, and relaxation—while enhancing accessibility, engagement, and reach [16,17].

A growing body of evidence supports the efficacy of iCBT for youth anxiety across diverse populations and settings [18,19,20,21,22]. However, while adolescent-focused iCBT programs are increasingly available, interventions tailored to younger children under 14 years remain limited and insufficiently tested. To enable translation of iCBT from research into routine clinical practice, robust evaluation is required not only of clinical outcomes but also of usability, engagement, and safety. Usability testing, emphasized in digital health frameworks, is a crucial developmental stage to ensure interventions are age-appropriate, acceptable, and technically reliable before larger-scale trials and clinical integration [12].

The present study aims to describe the development of a novel Italian iCBT software platform specifically tailored for children with mild to moderate anxiety symptoms and to report preliminary findings on its usability and safety through a pilot evaluation involving children and their caregivers.

## 2. Methods

### 2.1. Software Platform Development

The iCBT software platform was developed on the basis of DataRiver’s (Modena, Italy) MyHealth platform, originally designed for patient-centered clinical trials and previously used across multiple research fields. The iCBT platform was designed in compliance with international standards and guidelines for clinical trials (ICH GCP, 21 CFR part 11), cybersecurity (OSSTMM, OWASP, NIST 800-115, ISO-IEC 27000:2016, ISO/IEC 27001:2017, ISO/IEC 27002:2013), and the GDPR framework. It was released under Computer System Validation and hosted on a UNI EN ISO 27001-certified server farm to ensure physical, logical, and organizational information security.

The platform consists of 24 iCBT modules targeting both children and parents. The child component includes 12 modules with an empathetic interface and the following interactive features:

- Avatar-guided sessions;
- Audio recordings;
- Drawing tasks (e.g., representing an emotion);
- Open- and closed-ended questions;
- Image selection activities;
- Text-to-image linking exercises;
- Automated tracking of estimated completion time.

Modules incorporate psychoeducation, relaxation strategies, and cognitive restructuring, complemented by gamification elements such as badges, avatars, and motivational messages, consistent with best practices in digital mental health design [21]. To foster engagement, each module is interactive and supported by visual content, reminders, and short instructional videos.

Development followed a co-design approach that actively involved children, caregivers, clinicians, and digital health experts. A secure SMS reminder service was also implemented to encourage parental participation by prompting weekly responses to assigned modules.

Patient data are stored in an encrypted and segregated database, with multiple security layers including restricted access, firewalls, HTTPS connections, and disk encryption to ensure confidentiality both at rest and in transit. Automatic backups are performed regularly to guarantee data integrity, accuracy, and recovery. For intellectual property protection, the software was formally deposited with the Benelux Office for Intellectual Property (i-DEPOT n. 152570, 10 June 2025).

### 2.2. Pilot Study on Usability

Children aged 8–12 years presenting with mild to moderate anxiety symptoms, as indicated by a Multidimensional Anxiety Scale for Children–Second Edition (MASC-2) T-score between 55 and 64, were eligible to participate. Additional inclusion criteria included sufficient Italian language comprehension, regular access to an electronic device (tablet or computer), and the presence of at least one caregiver able to participate in the usability session. Exclusion criteria were the presence of chronic medical or neurological conditions, diagnosed psychiatric disorders requiring ongoing treatment, concurrent psychological or pharmacological therapy, or any condition that could interfere with participation (e.g., cognitive impairment, visual or hearing deficits).

Five families were recruited, each including a child aged 9–12 years with recent anxiety symptoms and no chronic medical or psychiatric comorbidities. Eligibility was first assessed by primary care pediatricians using the MASC-2. Children scoring within the moderate anxiety range (T-score 55–64) were invited, with their caregivers, for a confirmatory evaluation by a child psychiatrist at the Pediatric Clinic of the University Hospital of Parma. This clinical interview verified inclusion and exclusion criteria and assessed suitability for an internet-delivered intervention. The study was approved by the Ethics Committee of the University of Parma. Written informed consent was obtained from both children and their parents after a full explanation of study objectives and procedures.

Each child participated in a single supervised session in a quiet environment, following brief researcher-led training. This simulated “first-session” experience aimed to assess usability under controlled conditions. Participants were not expected to complete the full 12-week program; instead, they engaged with a selected portion of content to allow evaluation of navigation, comprehension, and user engagement.

Usability was assessed through direct observation, structured interviews with children and caregivers, and the Italian version of the mHealth App Usability Questionnaire (ita-MAUQ), validated by Podda et al. [23]. This phase corresponded to stage 3 (“usability and pilot testing”) of the co-design framework described by Hill et al. [24]. At the end of the session, participants were debriefed and reminded of their right to withdraw at any time.

The primary outcome was usability, measured through ita-MAUQ scores. Secondary outcomes included caregiver and child satisfaction, as well as spontaneously reported usability issues or negative experiences. For subsequent larger trials, exploratory outcomes will also include child quality of life, caregiver burden, and WebApp usage metrics.

### 2.3. Data Analysis

Descriptive statistics were computed for each ita-MAUQ domain and item, including means, standard deviations (SD), and frequency distributions. Open-ended responses and interview transcripts underwent thematic analysis. Although an electronic case report form (eCRF) system was prepared for future large-scale trials, data from this pilot study were recorded and analyzed locally and not entered into the eCRF. Qualitative data were collected through semi-structured interviews and open-ended questions administered immediately after the supervised usability session. Both children and caregivers were asked about their experiences with the WebApp, including ease of use, engagement, clarity of content, and suggestions for improvement. Interviews were audio-recorded when permitted and transcribed verbatim. Data were analyzed using an inductive thematic approach to identify recurring patterns and usability themes. Two researchers (M.R.P. and C.P.) independently reviewed and coded the transcripts, resolving discrepancies through discussion and consensus. Owing to the small sample size, the analysis was primarily descriptive and exploratory, intended to highlight key usability strengths and potential areas for refinement rather than to establish formal inter-rater reliability. Children’s and caregivers’ responses were analyzed separately to capture distinct perspectives: children’s feedback informed conclusions on interface design, interactivity, and game elements, whereas caregivers’ input contributed to understanding perceived usefulness, parental involvement, and overall satisfaction.

## 3. Results

### 3.1. Software Platform Overview

**Figure 1:**
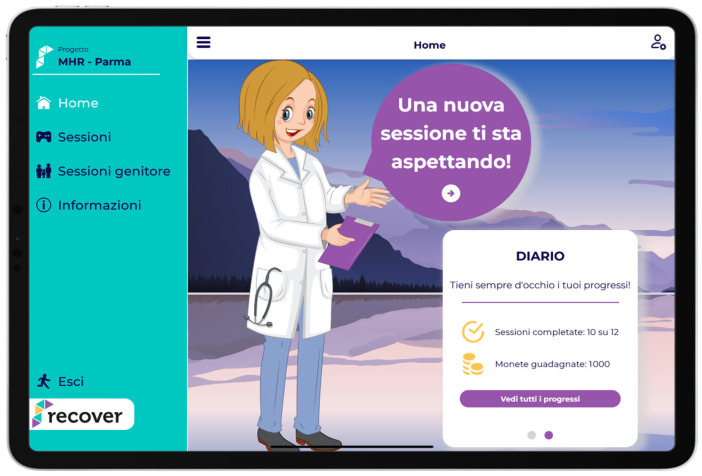
Overview of the iCBT software platform interface. The platform provides interactive activities aimed at engaging children and caregivers in managing anxiety symptoms. English translation: Home, Sessions, Sessions for parents, Information, Exit; A new session is waiting for you; Diary, Always keep track of your progress, Sessions completed: 10 of 12, Coins earned: 1000, View all progress.

The software platform allowed to engage families in a simple and fun way through low- and medium-impact family engagement activities, as well as with conversation starters on the child’s emotions, thoughts and actions to foster conversations both between the specialist and the child but especially between family members themselves (Figure 1).

**Figure 2:**
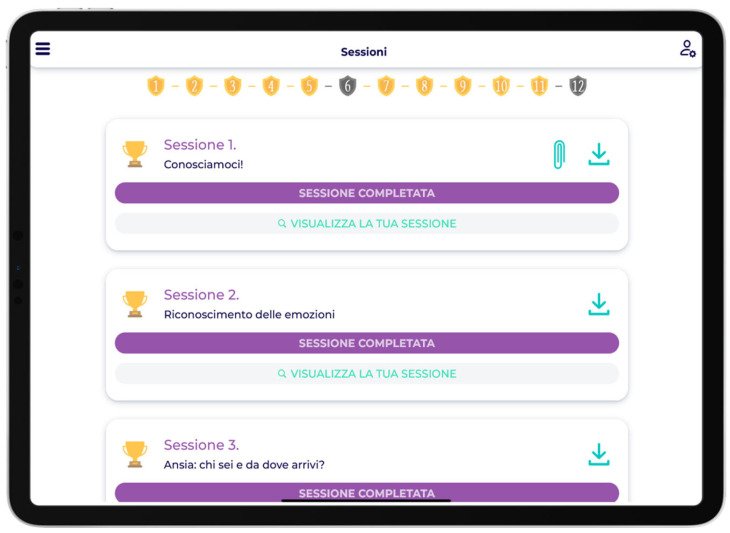
Dashboard view of session progress. Families could monitor completed, ongoing, or pending sessions through list and chart visualizations. English translation: Session 1, Let’s get to know each other!, SESSION COMPLETED, View your session; Session 2, Recognizing emotions, SESSION COMPLETED, View your session; Session 3, Anxiety: who are you and where do you come from?, SESSION COMPLETED, View your session.

Users with their own tablet/PC were able to monitor the status of all sessions on the completed, started or unblocked sessions list page or via a more intuitive chart on the home page (Figure 2).

The badges on the sessions page are an example of how the platform was designed, focusing on maximum child engagement.

**Figure 3:**
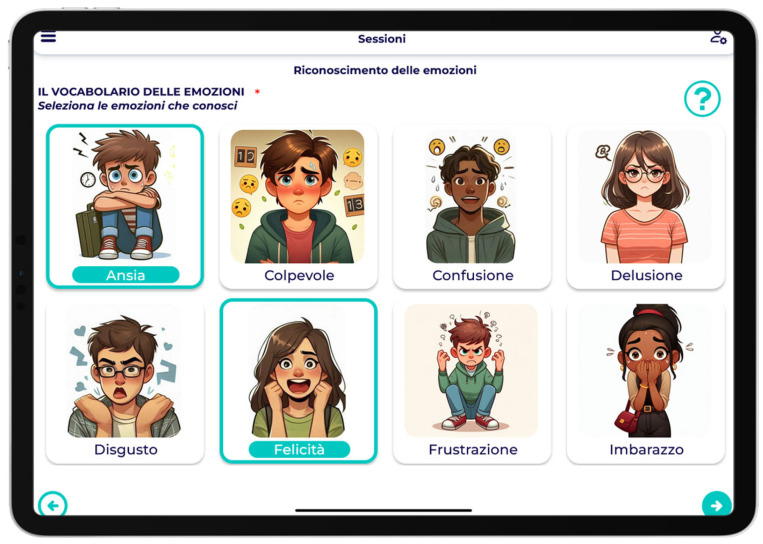
Example of image-selection task. Children were invited to express emotions, thoughts, or behaviors by selecting one or more pictures. English translation: Select the emotions you know: Emotion labels: Anxiety; Guilt; Confusion; Disappointment; Disgust; Happiness; Frustration; Embarrassment.

Among the activities proposed in the sessions, the user found himself expressing his thoughts, emotions and actions through multiple and non-multiple selection of images (Figure 3).

**Figure 4:**
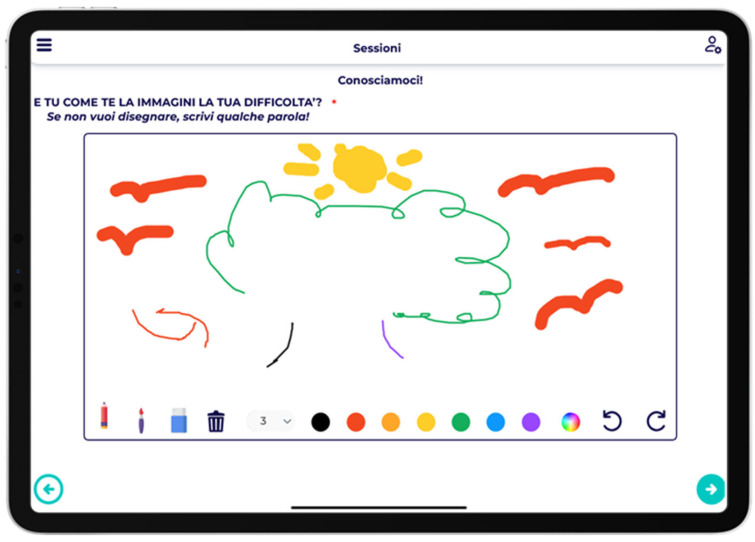
Drawing activity. The platform allowed children to freely express emotions and feelings using digital drawing tools and colors. English translation: AND HOW DO YOU IMAGINE YOUR DIFFICULTY? If you don’t want to draw, write a few words!

The platform also turns into a real blank sheet of paper where users can express their emotions and feelings through the strokes of the tools and colours provided (Figure 4).

**Figure 5:**
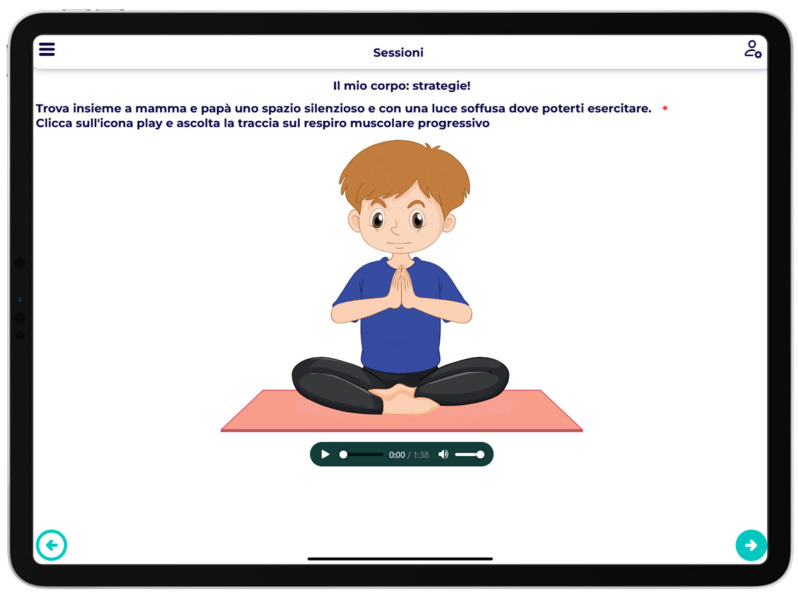
Audio-guided relaxation exercise. Listening to therapists’ voices supported children in coping with anxiety and strong emotions. English translation: My body: strategies! Find together with mom and dad a quiet space with soft light where you can practice. Click on the play icon and listen to the track on progressive muscle breathing.

Listening to the voice of therapists can help in finding a solution to cope with moments of anxiety and strong emotions to the best of one’s ability (Figure 5).

### 3.2. Usability Study Findings

Table 1 shows the characteristics of the study population. Five children (4 girls and 1 boy) aged between 9 and 12 years participated in the study, each accompanied by at least one caregiver. All children presented with mild to moderate anxiety, with MASC-2 T-scores ranging from 55 to 64. Families represented diverse household structures, including both single-parent and two-parent households. All participants successfully completed the supervised usability session without dropouts.

**Figure 6:**
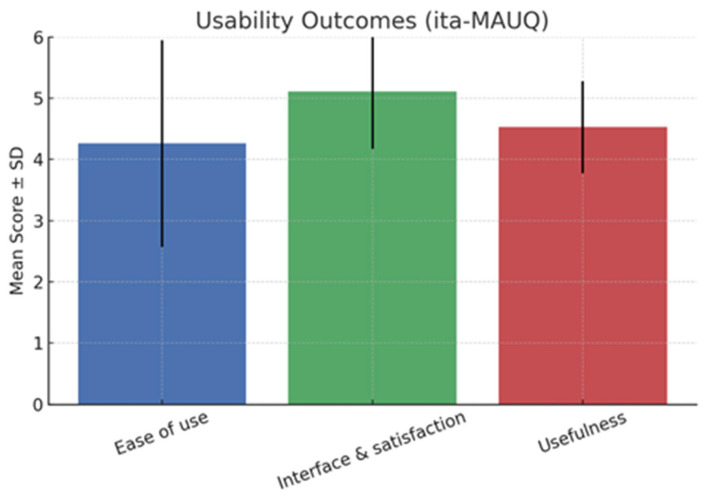
Usability outcomes (ita-MAUQ). Bars represent mean scores for each domain, with error bars showing standard deviation (SD). Exact mean ± SD values are displayed above each bar.

Figure 6 summarizes usability outcomes based on the ita-MAUQ. Overall, findings indicated positive acceptance of the digital tool, with mean scores consistently above the scale midpoint. The ease-of-use domain yielded a mean score of 4.26 (SD = 1.69), suggesting that most participants found the WebApp intuitive to navigate and its functions accessible. Although some variability was observed, the majority of children and caregivers reported being able to operate the WebApp without requiring extensive instruction, supporting its feasibility in pediatric use. The interface and satisfaction domain received the highest evaluation, with a mean of 5.11 (SD = 0.94). This reflects strong appreciation for the WebApp’s visual design, layout, and overall user-friendliness. The low variability further demonstrates consistent satisfaction across families. The usefulness domain scored a mean of 4.53 (SD = 0.75), indicating that participants perceived the WbApp as helpful and relevant for managing anxiety symptoms. The relatively narrow distribution of scores suggests that this perception was widely shared.

Table 2 shows the caregiver’s comments on the platform.

Importantly, no adverse psychological or technical events were reported during the testing phase, underscoring both the safety and acceptability of the app WebApp in this age group. Moreover, MASC-2 T-scores improved in all participants, with results <55 in the five patients after iCBT.

## 4. Discussion

This pilot study provides initial evidence on the usability and safety of a newly developed iCBT software platform specifically designed for children with mild to moderate anxiety symptoms. Usability testing is a critical step in the development of DTx, ensuring that interventions are acceptable, understandable, and engaging for their intended users before progressing to large-scale clinical trials [14]. By combining structured observation, semi-structured interviews, and a validated usability scale (ita-MAUQ), this study employed a multi-method strategy that strengthened both the reliability and depth of the usability assessment.

Overall, the WebApp was well accepted by children and caregivers. The intuitive interface and gamified elements—such as avatars, badges, and motivational prompts—were highlighted as particularly engaging features. These findings are in line with previous evidence showing that gamification can improve user motivation, adherence, and treatment retention in pediatric digital mental health interventions [24,25,26,27]. Importantly, no adverse psychological or technical events were reported, supporting the tolerability and safety of the platform in this sensitive age group.

Qualitative feedback proved essential for iterative refinement. Children and caregivers suggested improvements in navigation flow, language clarity, and further tailoring of content to developmental stages. The dual-user structure, which provides parallel modules for both children and caregivers, was especially valued. This feature emphasizes the role of family involvement in pediatric anxiety treatment, as caregiver engagement is known to strengthen therapeutic outcomes by promoting consistency, discouraging avoidance, and modeling adaptive coping strategies [25].

The findings carry broader implications for the design and implementation of pediatric DTx. First, they demonstrate the feasibility of translating evidence-based CBT principles into an interactive and child-friendly format accessible beyond traditional clinical settings. Second, the co-design process—actively involving children, caregivers, clinicians, and digital health experts—appears central to developing tools that are both clinically robust and developmentally appropriate. Third, the use of a secure, regulatory-compliant platform illustrates how early-phase digital therapeutic development can align with international standards of safety, privacy, and usability, which are prerequisites for clinical integration and eventual reimbursement.

The empirical findings align closely with the platform’s conceptual goals of improving access and promoting engagement in pediatric anxiety treatment. The intuitive, game-based interface and dual-user structure were positively received by both children and caregivers, supporting the platform’s potential to enhance motivation and sustained participation—key components of digital engagement. Moreover, the web-based delivery model directly addresses accessibility barriers, including geographic distance and limited availability of trained clinicians, thereby supporting broader reach within the healthcare system. Together, these findings suggest that the platform’s design successfully operationalized its foundational goals, offering a promising pathway toward more equitable, engaging, and scalable mental health interventions for children.

This study offers several novel contributions to the field of pediatric digital mental health. First, it represents one of the few Italian-language iCBT platforms specifically designed for younger children aged 8–12 years, a group often underrepresented in digital CBT research. Second, the platform incorporates a dual-user structure that actively engages both the child and caregiver, promoting family involvement—a distinctive element rarely emphasized in early-phase iCBT development. Third, the software was created following rigorous standards for clinical data security and regulatory compliance, positioning it within the emerging category of digital therapeutics rather than general wellness applications. Finally, by combining co-design principles with structured usability testing, this pilot establishes a reproducible model for developing and validating culturally adapted iCBT tools for children.

Recent studies have confirmed that iCBT represents a feasible and effective intervention for anxiety disorders in children and adolescents. Meta-analyses and systematic reviews have demonstrated moderate to large effects compared with waitlist or minimal-support conditions [17,18,19]. More recent investigations have extended this evidence to real-world and community-based settings, confirming that iCBT can be implemented effectively within youth mental health services [20,21]. Our study adds to this literature in several ways. First, it targets a younger age range (8–12 years), which remains underrepresented in digital CBT research and was highlighted as a key evidence gap in recent reviews [19,22]. Second, the platform integrates a dual-user structure involving both children and caregivers, consistent with parent-supported iCBT approaches such as the Online Support and Intervention (OSI) program developed in the United Kingdom [24]. This design promotes family engagement and may enhance treatment adherence. Finally, our use of a co-design framework and adherence to data security and regulatory standards align with emerging best practices in digital therapeutic development [13,14,28]. Together, these features underscore the novelty of this study and its contribution to advancing equitable, accessible, and developmentally tailored digital interventions for child anxiety.

Several limitations should be acknowledged. The pilot involved only five families, which restricts the generalizability of the findings. A larger and more diverse sample across different socioeconomic, cultural, and geographic contexts will be necessary to validate the platform’s usability and safety more robustly. The study’s focus on a single supervised session also limits insight into long-term engagement and real-world usage patterns. Multi-session testing in home and school environments, as suggested, would provide a more comprehensive evaluation of sustained adherence and user experience. All participants were Italian-speaking and recruited from a single site, which constrains the cultural and demographic applicability of the findings and underscores the need for future translation and cross-cultural validation. Moreover, although this feasibility study primarily assessed usability and safety, it did not evaluate clinical efficacy—such as reductions in anxiety symptoms—which is essential for establishing therapeutic effectiveness and supporting broader implementation. The absence of a waitlist or standard-care comparison group further limits the interpretability of any trends in symptom change, reinforcing the need for future randomized controlled designs with appropriate comparators. Finally, the likelihood of an observer (Hawthorne) effect must be considered, as participants were aware of being observed, and self-reported satisfaction data may have been subject to bias [29,30]. Complementing self-reports with objective indicators such as platform usage logs would strengthen future evaluations of engagement and effectiveness.

Future research should therefore expand evaluation of this WebApp through larger randomized controlled trials, examining not only usability and safety but also clinical effectiveness, cost-effectiveness, and sustained engagement. Outcomes should be stratified by age, gender, digital literacy, and cultural background to ensure inclusivity. Incorporating adaptive features such as personalized feedback, progress tracking, and real-time clinician support could further strengthen therapeutic impact. Longitudinal studies will also be essential to determine whether early digital interventions yield lasting benefits throughout adolescence and into adulthood.

Looking ahead, future development of the iCBT platform will prioritize issues of equity, cultural adaptation, and inclusion. The current version was tested only with Italian-speaking families, and expanding accessibility will require translation, linguistic validation, and adaptation to diverse cultural contexts to ensure relevance across populations. Future studies will aim to include participants from varied socioeconomic and geographic backgrounds, exploring barriers such as digital literacy and access to technology that may affect equitable use. Incorporating inclusive design principles—such as customizable content, flexible language options, and culturally sensitive examples—will further support usability and engagement among diverse groups. These efforts are essential to ensure that the platform contributes to reducing disparities in access to evidence-based mental health care for children.

## 5. Conclusions

Although still in the experimental phase, this newly developed iCBT software platform represents a meaningful contribution to the growing field of pediatric digital mental health. Designed and tested within the Italian context, and developed in compliance with evolving national and European regulatory frameworks for DTx, the platform demonstrates that early-phase usability testing can provide critical insights for iterative refinement while ensuring adherence to international standards of safety, privacy, and quality. The findings of this pilot study support the feasibility, acceptability, and preliminary safety of delivering CBT through a digital, child-friendly interface that also actively involves caregivers. At the same time, the exclusive testing with Italian-speaking families underscores the importance of future cross-cultural validation and multilingual adaptation to ensure broader applicability. Taken together, these results lay the groundwork for larger-scale trials aimed at evaluating clinical effectiveness, long-term engagement, and cost-effectiveness. Ultimately, this research supports international efforts to develop evidence-based, scalable, and developmentally appropriate digital interventions that can improve access to care for children with anxiety disorders.

## Figures and Tables

**Table 1:** Characteristics of the study population.

Patient Identification Number	Age, Years	Gender	Nationality	Anxiety Level, MASC-2 T-Scores	Participating Caregiver, Age and Educational Qualification
1	10	F	Italian	60	Mother, 44 y, degree in physiotherapy, two children
2	12	M	Italian	58	Mother, 46 y, technical institute diploma, one child
3	9	F	Italian	64	Father, 38 y, degree in dentistry, two children
4	9	F	Italian	57	Mother, 38 y, technical institute diploma, one child
5	12	F	Italian	55	Mother, 44 y, degree in nursing, PhD in public health, two children

**Table 2:** Caregiver’s comments on the platform.

Patient	Caregiver’s Comments
1	The content is very easy and clear to follow. The strategy-focused sections are useful and practical.
2	The section dedicated to parents is truly invaluable in trying to support and not hinder my son’s difficulties.
3	On a technical level, since we’re not very technologically savvy, it was a bit complex at times, but my daughter was thrilled to do the modules because she found them very useful.
4	This app would be a great “buffer” for those who remain on the waiting list for public services.
5	The app is very useful, because there are easily applicable strategies.

## Data Availability

The original contributions presented in this study are included in the article. Further inquiries can be directed to the corresponding author.

## References

[1] Kowalchuk A., Gonzalez S.J., Zoorob R.J. (2022). Anxiety Disorders in Children and Adolescents. Am. Fam. Physician.

[2] Gao S., Chen Y., Li Y., Li Y., Yin A., Yan M., Zhou L., Luo L. (2025). Social Feedback Perception in Adolescents with Major Depressive Disorder: A Potential Marker of Depression. Early Interv. Psychiatry.

[3] Wang W., Zhu W., Yuan H., Liu L., Wu X. (2025). Gender differences for the relationships between posttraumatic stress disorder, depression, and anxiety in postearthquake adolescents: A panel graphical vector autoregression model. Psychol. Trauma.

[4] Esposito S., Campana B.R., Argentiero A., Masetti M., Fainardi V., Principi N. (2025). Too young to pour: The global crisis of underage alcohol use. Front. Public Health.

[5] Apicella M., Andracchio E., Della Santa G., Lanza C., Guidetti C., Trasolini M., Iannoni M.E., Maglio G., Vicari S., Serra G. (2025). Sex differences in pediatric major depressive episodes: A cross-sectional study on psychiatric symptoms in early-onset mood disorders. Front. Psychiatry.

[6] Kim D. (2025). The Effects of Child Mental Health on Juvenile Criminal Justice Contact and Victimization. J. Ment. Health Policy Econ..

[7] Casey B.J., Glatt C.E., Lee F.S. (2015). Treating the Developing versus Developed Brain: Translating Preclinical Mouse and Human Studies. Neuron.

[8] Green J.G., McLaughlin K.A., Berglund P.A., Gruber M.J., Sampson N.A., Zaslavsky A.M., Kessler R.C. (2010). Childhood adversities and adult psychiatric disorders in the national comorbidity survey replication I: Associations with first onset of DSM-IV disorders. Arch. Gen. Psychiatry.

[9] Radez J., Reardon T., Creswell C., Lawrence P.J., Evdoka-Burton G., Waite P. (2021). Why do children and adolescents (not) seek and access professional help for their mental health problems? A systematic review of quantitative and qualitative studies. Eur. Child Adolesc. Psychiatry.

[10] Piacentini J., Bennett S., Compton S.N., Kendall P.C., Birmaher B., Albano A.M., March J., Sherrill J., Sakolsky D., Ginsburg G. (2014). 24- and 36-week outcomes for the Child/Adolescent Anxiety Multimodal Study (CAMS). J. Am. Acad. Child Adolesc. Psychiatry.

[11] Stewart R.E., Chambless D.L. (2009). Cognitive-behavioral therapy for adult anxiety disorders in clinical practice: A meta-analysis of effectiveness studies. J. Consult. Clin. Psychol..

[12] Hill C., Creswell C., Vigerland S., Nauta M.H., March S., Donovan C., Wolters L., Spence S.H., Martin J.L., Wozney L. (2018). Navigating the development and dissemination of internet cognitive behavioral therapy (iCBT) for anxiety disorders in children and young people: A consensus statement with recommendations from the #iCBTLorentz Workshop Group. Internet Interv..

[13] Batastini A.B., Paprzycki P., Jones A.C.T., MacLean N. (2021). Are videoconferenced mental and behavioral health services just as good as in-person? A meta-analysis of a fast-growing practice. Clin. Psychol. Rev..

[14] Perry K., Gold S., Shearer E.M. (2020). Identifying and addressing mental health providers’ perceived barriers to clinical video telehealth utilization. J. Clin. Psychol..

[15] Seuling P.D., Fendel J.C., Spille L., Göritz A.S., Schmidt S. (2024). Therapeutic alliance in videoconferencing psychotherapy compared to psychotherapy in person: A systematic review and meta-analysis. J. Telemed. Telecare.

[16] James A.C., Reardon T., Soler A., James G., Creswell C. (2020). Cognitive behavioural therapy for anxiety disorders in children and adolescents. Cochrane Database Syst. Rev..

[17] Vigerland S., Lenhard F., Bonnert M., Lalouni M., Hedman E., Ahlen J., Olén O., Serlachius E., Ljótsson B. (2016). Internet-delivered cognitive behavior therapy for children and adolescents: A systematic review and meta-analysis. Clin. Psychol. Rev..

[18] Pennant M.E., Loucas C.E., Whittington C., Creswell C., Fonagy P., Fuggle P., Kelvin R., Naqvi S., Stockton S., Kendall T. (2015). Computerised therapies for anxiety and depression in children and young people: A systematic review and meta-analysis. Behav. Res. Ther..

[19] Andrews G., Cuijpers P., Craske M.G., McEvoy P., Titov N. (2010). Computer therapy for the anxiety and depressive disorders is effective, acceptable and practical health care: A meta-analysis. PLoS ONE.

[20] Arnberg F.K., Linton S.J., Hultcrantz M., Heintz E., Jonsson U. (2014). Internet-delivered psychological treatments for mood and anxiety disorders: A systematic review of their efficacy, safety, and cost-effectiveness. PLoS ONE.

[21] Richards D., Timulak L., Rashleigh C., McLoughlin O., Colla A., Joyce C., Doherty G., Sharry J., Duffy D., Anderson-Gibbons M. (2016). Effectiveness of an internet-delivered intervention for generalized anxiety disorder in routine care: A randomised controlled trial in a student population. Internet Interv..

[22] Willutzki U., Teismann T., Schulte D. (2012). Psychotherapy for social anxiety disorder: Long-term effectiveness of resource-oriented cognitive-behavioral therapy and cognitive therapy in social anxiety disorder. J. Clin. Psychol..

[23] Podda J., Grange E., Susini A., Tacchino A., Di Antonio F., Pedullà L., Brichetto G., Ponzio M. (2024). Italian Version of the mHealth App Usability Questionnaire (Ita-MAUQ): Translation and Validation Study in People With Multiple Sclerosis. JMIR Hum. Factors.

[24] Hill C., Reardon T., Taylor L., Creswell C. (2022). Online Support and Intervention for Child Anxiety (OSI): Development and Usability Testing. JMIR Form. Res..

[25] Fleming T.M., Bavin L., Stasiak K., Hermansson-Webb E., Merry S.N., Cheek C., Lucassen M., Lau H.M., Pollmuller B., Hetrick S. (2017). Serious Games and Gamification for Mental Health: Current Status and Promising Directions. Front. Psychiatry.

[26] Cerrillo-Urbina A.J., García-Hermoso A., Sánchez-López M., Pardo-Guijarro M.J., Santos Gómez J.L., Martínez-Vizcaíno V. (2015). The effects of physical exercise in children with attention deficit hyperactivity disorder: A systematic review and meta-analysis of randomized control trials. Child Care Health Dev..

[27] Arnett A.B., Fearey M., Peisch V., Levin A.R. (2022). Absence of dynamic neural oscillatory response to environmental conditions marks childhood attention deficit hyperactivity disorder. J. Child Psychol. Psychiatry.

[28] Scholten H., Granic I. (2019). Use of the Principles of Design Thinking to Address Limitations of Digital Mental Health Interventions for Youth: Viewpoint. J. Med. Internet Res..

[29] McCambridge J., Witton J., Elbourne D.R. (2014). Systematic review of the Hawthorne effect: New concepts are needed to study research participation effects. J. Clin. Epidemiol..

[30] Grist R., Croker A., Denne M., Stallard P. (2019). Technology Delivered Interventions for Depression and Anxiety in Children and Adolescents: A Systematic Review and Meta-analysis. Clin. Child Fam. Psychol. Rev..

